# Enslaved ants: not as helpless as they were thought to be

**DOI:** 10.1007/s00040-014-0377-z

**Published:** 2014-12-17

**Authors:** W. Czechowski, E. J. Godzińska

**Affiliations:** 1 Laboratory of Social and Myrmecophilous Insects, Museum and Institute of Zoology, Polish Academy of Sciences, Wilcza 64, 00-679 Warsaw, Poland; 2 Laboratory of Ethology, Department of Neurophysiology, Nencki Institute of Experimental Biology, Polish Academy of Sciences, Pasteura 3, 02-093 Warsaw, Poland

**Keywords:** Ants, Dulosis, Social parasitism, Mixed colonies, Slave rebellion, Slave emancipation

## Abstract

Slavery in ants involves robbing of brood of host ant species and rearing captured individuals in the enslaver’s nest. Whereas slaves of facultative slave-makers increase the workforce of the colony, in obligate slave-makers presence of slaves is vital for colony survival. Until recently, it was generally believed that enslaved workers act solely for the benefit of their social parasite and are wholly lost for their own colony and population. However, evidence that slaves may act also in favour of their own maternal population by engaging in various forms of the so-called slave rebellions is already quite extensive and may be found in both old and recent myrmecological literature, although, unfortunately, these data are often neglected or overlooked. They may be classified into four categories: (1) acts of physical aggression directed by slaves to slave-makers, (2) attempts of slaves to reproduce within a slave-maker colony, (3) ‘sabotage’, i.e. activities of slaves leading to weakening of the slave-maker colony and population, and (4) slave emancipation, i.e. partial or complete self-liberation of slaves from slave-maker colonies. In this review, we present and discuss all these diverse (often interrelated) expressions of slave opposition to their enslavers, focussing our discussion on both proximate and evolutionary causation of the discussed phenomena. We also indicate some open questions which remain to be answered by future research.

## Introduction

Slavery (dulosis) is the most spectacular form of social parasitism in ants and one of the most unusual interspecific relationships in the animal world (e.g. Wheeler, 1910; Forel, 1928; Buschinger, 1990, 2009; Hölldobler and Wilson, 1990; D’Ettorre and Heinze, 2001). Slave-makers rob brood from colonies of their host species and transport them to their own nests. As a consequence, adult workers of the host species, the so-called slaves, emerge in the enslaver’s nest and subsequently recognise it—due to an early olfactory learning process (Le Moli, 1980; Le Moli and Mori, 1985, 1987a, b; Blatrix and Sermage, 2005)—as their own and act for the benefit of the social parasite’s colony (e.g. Wheeler, 1910; Dobrzański, 1965; Wilson, 1975a; Buschinger et al., 1980; Hölldobler and Wilson, 1990; D’Ettorre and Heinze, 2001; Mori et al., 2001; Buschinger, 2009).

Slavery can be facultative or obligate. Colonies of facultative slave-makers, such as some species of the subgenus *Raptiformica*, may function successfully without slaves (e.g. Dlusskij, 1967; Marikovskij, 1967; Savolainen and Deslippe, 1996a; Deslippe, 2010), but slaves further contribute to their ecological and evolutionary success by increasing their workforce (Savolainen and Deslippe, 1996b). As demonstrated for the facultative slave-maker *Formica subnuda* (a Nearctic blood-red ant) and its slaves *F. podzolica*, additional energy contributed by slaves may be allocated to increase production of the enslaver sexual brood (Savolainen and Deslippe, 1996b). In the case of obligate slavery, presence of slaves is an essential condition for the survival of the colony, as abilities of enslaver workers to carry out everyday tasks are usually highly restricted, among others due to morphological and functional limitations (e.g. Beck, 1961; Kutter, 1969; Wilson, 1975b; Dobrzańska, 1978b; Buschinger et al., 1980; Mori and Le Moli, 1988; Buschinger, 2009; Szczuka et al., 2011). Moreover, workers of obligate social parasites often occur in limited numbers, whereas their slaves are relatively numerous (Wheeler, 1910; Talbot, 1967; Wilson, 1971; Hölldobler and Wilson, 1990; Savolainen and Deslippe, 1996a; Seifert, 2007; Buschinger, 2009).

Slavery is not a frequent phenomenon among ants. As estimated by D’Ettorre and Heinze (2001), there is not much more than 60 species of slave-making ants, i.e. as few as ca 0.5 % of all known extant ant species, and about one-fourth of all ant social parasites. Slavery has been discovered in six genera of Myrmicinae (*Chalepoxenus*, *Harpagoxenus*, *Protomognathus, Temnothorax*, *Myrmoxenus*, *Strongylognathus*) and three genera of Formicinae (*Formica*, *Polyergus*, *Rossomyrmex*). Whereas myrmicine slave-makers use as their hosts ant species of other myrmicine genera (*Leptothorax*, *Temnothorax*, *Tetramorium*; e.g. Buschinger et al., 1980; Hölldobler and Wilson, 1990), formicine slave-makers use some species of the genus *Formica* (in *Formica* and *Polyergus*; e.g. Buschinger et al., 1980; Hölldobler and Wilson, 1990) and *Proformica* (in *Rossomyrmex*; Zamora-Muñoz et al., 2003; Ruano et al., 2013).

Until recently, it was generally believed that individuals of a host species once enslaved serve the colony of their social parasite unconditionally, and are wholly lost for their own colony and population. It was even theoretically argued why so-called rebellions or revolts of enslaved individuals are impossible in evolutionary terms: slaves were supposed to be entirely unable to free themselves and reproduce (Gladstone, 1981). This belief was additionally strengthened by frequently observed cases of direct collaboration of slaves with their enslavers. In particular, slaves were repeatedly observed to fight actively with the ants attacked by their enslavers, including their conspecifics, and possibly even their former nestmates, and to participate in capture and transport of robbed brood. Such behaviour was described e.g. in *Temnothorax curvispinosus* slaves of *T. duloticus* (Wesson, 1937; Wilson, 1975b), *Formica fusca* and *F. cinerea* slaves of *Formica sanguinea* (Dobrzański, 1961; Czechowski, 1977, 1998), *Leptothorax acervorum* slaves of *Harpagoxenus sublaevis* (Buschinger and Winter, 1977; Winter, 1979a), *Temnothorax unifasciatus* slaves of *Myrmoxenus ravouxi* (Winter, 1979b), *Temnothorax ambiguus* and *T. longispinosus* slaves of *Protomognathus americanus* (Alloway, 1979; Alloway and Del Rio Pesado, 1983), and *Tetramorium* spp. slaves of dulotic *Strongylognathus* spp. (Sanetra and Buschinger, 1996). In colonies of *Strongylognathus testaceus*, the so-called degenerate slave-maker, with a vanishing ability to raiding *Tetramorium* c.f. *caespitum*, host workers themselves—and not workers of the social parasite—were observed to attack their free conspecifics and rob pupae (Forel, 1874). Similar phenomena were observed in colonies of *Protomognathus americanus* with *Temnothorax longispinosus* slaves (Alloway and Del Rio Pesado, 1983).

Dobrzańska (1978a) reported numerous observations of well-organised cooperation of slaves of *Polyergus rufescens* (a Euro-Siberian amazon ant) with their enslavers during the raids. *Formica cinerea* slaves were frequently seen to pick up pupae abandoned by *P. rufescens* on the nest. They also responded to *P. rufescens* returning from a raid by rushing toward them, snatching from them pupae, and retrieving the pupae to the nest. *Formica fusca* slaves were also observed to pick up pupae dropped by *P. rufescens* close to the nest entrance, but less frequently than *F. cinerea.* They collaborated with *P. rufescens* mostly by uncovering and enlarging nest openings to facilitate the mass entry of amazon ants returning from the raid. Picking up of booty abandoned by *P. rufescens* at the nest entrance was described also in *F. cunicularia* (Mori et al., 1991a, b). *Formica* *fusca* slaves from colonies of *P. rufescens* were also observed to organise their own ‘mini raids’, following raiding columns of the enslaver and picking up host pupae abandoned around the attacked nest, overlooked by the raiders or lost on the way (Czechowski, 2007). Interestingly, *F. fusca* workers enslaved by *P. rufescens* lose their characteristic ‘mildness’, and become aggressive to a degree unusual for that species. They were, for instance, observed to fight fiercely on the side of *P. rufescens* with *F. sanguinea* which attacked their mixed colony (Dobrzańska and Dobrzański, 1960). Workers of wood ants (subgenus *Formica* s. str.) enslaved by *F. sanguinea* also readily participated in competitive (territorial) actions against neighbouring conspecific colonies, including direct fight with their free conspecifics at *F. sanguinea*’s side (Czechowski, 1989).

The strength of integration of slaves with the colony of their enslaver is well illustrated also by the observations of the so-called nestmate rescue behaviour, i.e. coming to the rescue of a nestmate facing a dangerous situation. Czechowski et al. (2002) described that behaviour in workers of *Formica fusca* enslaved by *F. sanguinea* coming at the rescue of their *F. sanguinea* nestmates captured by ant lion larvae. Interestingly, *F. fusca* did not rescue their homospecific nestmates, and were themselves not rescued by *F. sanguinea*. Workers from monospecific colonies of *F. fusca* also did not come to the rescue of nestmates trapped by ant lion larvae.

The conviction about absolute ‘loyalty’ of slaves to their enslavers was so well established that the title of a recent work reporting a finding of the contrary phenomenon starts with a phrase “First evidence for slave rebellion…” (Achenbach and Foitzik, 2009). However, extensive and multifold evidence concerning various forms of the acts of opposition of slaves to slave-makers can be found even in very early myrmecological literature, not to mention numerous facts reported in more recent papers. In this review, all phenomena which may fall within the term ‘slave rebellions’ (or ‘revolts’) have been classified into four main categories: (1) ‘domestic misunderstandings’: episodes of physical aggression of slaves directed to slave-maker adults, (2) slave reproduction: attempts of slaves to reproduce within a slave-maker colony, (3) ‘sabotage’: activities of slaves leading to weakening of a slave-maker colony and/or population, and (4) slave emancipation: partial or complete self-liberation of slaves from a slave-maker colony.

## Survey of various categories of slave rebellions

### Domestic misunderstandings

Agonistic interactions involving more or less escalated overt aggression between slave-making ants and their slaves were observed (all under laboratory conditions) in mixed colonies of, chronologically, *Temnothorax duloticus* (Wilson, 1975b), *Protomognathus americanus* (Alloway and Del Rio Pesado, 1983) and *Harpagoxenus sublaevis* (Heinze et al., 1994), all obligate myrmicine slave-makers, and their slaves of the genera *Temnothorax* (the first two of the mentioned slave-makers) and *Leptothorax* (the last one).

Such ‘domestic misunderstandings’ are especially common in mixed colonies of the Palaearctic slave-maker *Harpagoxenus sublaevis*. In this species, intra-colony relations are characterised by frequent skirmishes among slave-maker workers themselves, between slave-makers and slaves, and among slaves of both the same and different species (when enslaved workers of two host species, *Leptothorax acervorum* and *L. muscorum*, occur in the same nest) (Heinze et al., 1994). Out of these two slave species, *L. muscorum* appeared to be more aggressive towards *H. sublaevis* in colonies in which these slaves considerably outnumbered *L. acervorum*. In such colonies, *L. muscorum* spontaneously attacked their *H. sublaevis* enslavers. In other colonies, acts of aggression of slaves against slave-maker workers were sporadic and usually happened after a slave had been attacked by an enslaver. During such attacks, slaves usually assumed a submissive posture. However, sometimes they were observed to catch hold of and/or pull a leg or an antenna of the opponent. There is no information on possible victims and/or body injuries of participants of these intra-colony ‘misunderstandings’ between allospecific nestmates, so it seems that these agonistic interactions were relatively unescalated and did not lead to serious injuries.

In contrast to *Harpagoxenus sublaevis*, in colonies of the Nearctic slave-maker *Protomognathus* *americanus*, enslavers were not observed to attack slaves, but slaves (*Temnothorax ambiguus* and *T. longispinosus*) were frequently observed to bite *P. americanus* workers and to drag them out of the nest. These attacks were relatively escalated: as a result, some enslavers lost parts of their appendages. However, such hostile encounters always occurred as single events and never took on a form of a mass slave revolt. Interestingly, *P. americanus* workers attacked by a specific slave were cared for (fed and groomed) by other slaves, and a slave attacking one individual treated other *P. americanus* workers in a friendly way (Alloway and Del Rio Pesado, 1983).

Intra-colony duels between slave-maker workers, between slaves and between slaves and slave-makers were also observed in mixed colonies of *Temnothorax duloticus*, a Nearctic slave-maker which, similarly as *Protomognathus americanus*, uses ants of the genus *Temnothorax* (e.g. *T. curvispinosus*) as its slaves. Some of these incidents were violent and long-lasting, but never ended fatally. They were always initiated by slaves, and attacked slave-maker workers were never seen to fight back (Wilson, 1975b). Workers of *T. curvispinosus* were even observed to attack queens of *T. duloticus* (see “Slave reproduction”).

Internal conflicts within mixed colonies, due to their frequency and a high level of aggressiveness of slaves, may escalate into ‘sabotage’ and—at least under laboratory conditions—end disastrously for the slave-maker. Such cases were observed in some Nearctic *Polyergus* species and their slaves of the *Formica* *pallidefulva* species group (King and Trager, 2007) (see “Sabotage”).

### Slave reproduction

Worker reproduction is common in eusocial Hymenoptera (see e.g. Bourke, 1988a; Wenseleers et al., 2004) including ants (e.g. Peeters, 1991; Helanterä and Sundström, 2007), and among them dulotic species (e.g. Bourke et al., 1988; Herbers and Stuart, 1998; Foitzik and Herbers, 2001; Blatrix and Herbers, 2004; Brunner et al., 2005). Worker egg-laying is even especially intensive in slave-makers in comparison with non-parasitic ant species, as dulotic ants are more strongly selected to reproduce (see e.g. Hung, 1973; Wilson, 1975b; Winter and Buschinger, 1983; Heinze, 1996a, b). For example, in colonies of *Polyergus rufescens*, up to 100 % of males are produced by workers (Brunner et al., 2005). Similarly, in *Protomognathus americanus*, also an obligate slave-maker, worker reproduction accounts for more than 70 % of all males (Foitzik and Herbers, 2001).

Generally, in ants, worker oviposition starts or increases when the colony is orphaned (becomes queenless) which suggests self-restraint or self-policing of workers in queenright conditions and/or some control from the queen(s) (e.g. Mamsch, 1967; Bourke, 1988b; Czechowski, 1996; Helanterä and Sundström, 2007). Enslaved workers also may lay eggs in nests of their enslavers in which they are deprived from presence of homospecific queens. However, Czechowski’s (unpubl. data) intense search of *Formica fusca* males as expected sons of the slaves in colonies of *F. sanguinea* living under natural conditions ended in failure. Males of *F. fusca* were not found even in a *F. sanguinea* colony with an unusually high proportion (58 %) of slaves. The vast majority of *F. fusca* workers was found in the deepest parts of the nest (Czechowski, 1996) which suggests that in that colony slaves consisted predominantly of young individuals, predisposed to egg-laying. Ovaries of young ant workers are known to be relatively well developed and to regress as a function of individual age and behavioural specialisation (ovaries of extranidal workers are as a rule regressed) (e.g. Otto, 1958; Hohorst, 1972; Billen, 1982; Fresneau, 1984; Minkenberg and Petit, 1985; Tsuji, 1988; Fénéron et al., 1996). The colony under discussion was excavated in July 1992, transferred to laboratory and split into two parts, a queenright and a queenless one. The queenright part was initially composed of about 300 (37.5 %) workers of *F. sanguinea* and 500 (62.5 %) workers of *F. fusca*; the queenless one counted about 700 (64 %) workers of *F. sanguinea* and 400 (36 %) workers of *F. fusca*. During the same and the next season (1993), the queenright part was subjected to a series of adoption experiments (see Czechowski, 1996), whereas the queenless one was left alone (except an unsuccessful attempt to introduce *F. fusca* queens in May 1993; see also “Discussion/Open questions”). Slave males never appeared in the colony part with a *F. sanguinea* queen, while in the queenless part about 100 male larvae and pupae appeared in May 1993. Then, within ca 2 months, 53 *F. fusca* adult males emerged there. It is puzzling that no *F. sanguinea* males were observed in both cultures (Czechowski, 1996). A similar phenomenon was observed also by E.J. Godzińska and co-workers (unpubl. data) who transferred to the laboratory a large queenless fragment of a natural mixed colony of *F. sanguinea* and *F. fusca*. This time, too, no *F. sanguinea* males were produced, but after several months the mixed culture contained numerous *F. fusca* large males (macraners). All these findings suggest that at least in *F.* (*Raptiformica*) *sanguinea*, a Palaearctic facultative slave-maker, the production of males by *F. fusca* slaves is efficiently inhibited by the presence of the dulotic queen and starts when the queen is lost.

Hung (1973) obtained, however, opposite results for the Nearctic facultative slave-maker *Formica* (*Raptiformica*) *pergandei* and the obligate slave-maker *Polyergus breviceps*, and their slave species *Formica canadensis* (the *fusca* group). He created mixed queenless cultures composed of slave-maker workers and their slaves out of individuals taken from natural mixed colonies collected in the field. Production of slave males was fully inhibited in presence of enslaver workers even in absence of a slave-maker queen in both *F. pergandei* and *P. breviceps.* In such mixed cultures, only males of both slave-maker species were produced. In contrast, males of *F. canadensis* were produced in control colonies composed only of workers of that species. Similarly, workers of *F. japonica* enslaved by the Japanese amazon ant *Polyergus samurai* produced their own males only in absence of the slave-maker workers (Tsuneoka, 2006).

An interesting situation was observed in a colony of *Polyergus rufescens* with *Formica cinerea* slaves found in the Kampinos Forest (central Poland). One nest of the polydomous nest system of *F. cinerea* was occupied by *P. rufescens*. Nuptial flights of *F. cinerea* males (and only males) were observed to depart from all nest holes of that nest system with the exception of that single nest (or, more precisely, a group of nest holes) (Czechowski, 1975). If in that peculiar mixed colony queen(s) of *F. cinerea* were absent, these males must have been the sons of *P. rufescens* slaves. If so, this observation would imply that *F. cinerea* workers may reproduce only if they are physically separated from a *P. rufescens* queen. However, theoretically, according to the principle of oligogyny, a queen or queens of *F. cinerea* might have been present at a sufficient distance from the slave-maker queen and it cannot be excluded that the observed males were her sons (see “Slave emancipation”).

Yet another situation was observed under laboratory conditions in colonies of the obligate slave-maker *Harpagoxenus sublaevis.* Slaves of this ant species, *Leptothorax acervorum* and *L. muscorum*, reproduced even in queenright colonies of the enslaver. The majority of aggressive interactions between slave-maker workers and slaves within mixed colonies of these species (see “Domestic misunderstandings”) had for their background the competition for the production of males. The enslaver workers attempted to prevent the slaves from egg-laying by attacking and dominating them. Anyway, eggs laid by the slaves were usually destroyed by the enslaver or policed by other slaves (Heinze et al., 1994). Slave egg-laying was also reported, under laboratory conditions as well, in colonies of *Temnothorax duloticus*. Slaves of that species, *T. curvispinosus*, laid eggs directly on an egg pile guarded by the slave-maker queen. Before egg-laying, they frequently approached the queen and engaged in biting of her head or thorax. Once, a slave was observed to lay an egg at some distance from the queen, and then to insert it peacefully into the egg pile (Wilson, 1975b).

### Sabotage

Several laboratory studies reported that slaves may kill a large part of their enslaver’s brood. This phenomenon was described for the first time by Alloway and Del Rio Pesado (1983) in an incipient colony of *Protomognathus americanus* transferred to the laboratory from the field. The colony consisted of a slave-maker queen, her brood and some workers of *Temnothorax longispinosus*, remnants of an originally ‘free’ colony of that slave species, taken over by a *P. americanus* queen. Due to that, despite the fact that the authors used the term “slaves” to denote these workers of *T. longispinosis*, a more general term “hosts” seems to be more appropriate here. These host workers of *T. longispinosus* cared for slave-maker brood throughout the pupal instar, but then they killed all eclosing *P. americanus* workers. Apart from this own single observation, Alloway and Del Rio Pesado (1983) mentioned also that “similar events in other incipient *H.* [*Harpagoxenus* *=* *Protomognathus*] *americanus* colonies” have been observed (but not published) by R.J. Stuart.

More recently, Achenbach and Foitzik (2009) reported the same phenomenon (also under laboratory conditions) in *Temnothorax curvispinosus*, *T. longispinosus* and *T. ambiguus* enslaved by *Protomognathus americanus* (see also Pamminger et al., 2013). Slaves of these species systematically eliminated up to two-thirds of female brood of their enslaver. Worker and gyne pupae of *P. americanus* were either directly killed, or removed from the nest and abandoned. It should be stressed that these events took place in mature (not incipient) *P. americanus* colonies, in which *Temnothorax* workers were real slaves, and not remnants of originally free host species colonies taken over by slave-maker queens. The authors interpreted their findings in terms of an anti-parasite strategy acting as a second line of defence of the slave species against the slave-maker which may be adopted if direct defence of the slave colony ended in failure. Thanks to that strategy, slaves indirectly benefit local populations of their own species by decreasing enslaver’s workforce and colony fitness. Slower growth of slave-maker colonies reduces their raiding activity and, at the same time, their impact on neighbouring host colonies.

The question of proximate causal factors underlying this form of ‘sabotage’ (killing of slave-maker’s brood) also needs further research. In relation to slave rebellions within laboratory mixed colonies of *Protomognathus americanus* and their *Temnothorax* slaves, Achenbach et al. (2010) sought their basis in differential cuticular hydrocarbon profiles of the parasite and slave pupae. However, Johnson et al. (2005) showed that workers of two host species of the obligate slave-maker *Polyergus breviceps*, *Formica gnava* and *F. occulta*, are able to reject eggs of *P. breviceps* in spite of the fact that in terms of hydrocarbon profiles parasite eggs may be locally adapted to their host species. It should also be pointed out that killing of slave-maker brood by slave workers was so far observed only under laboratory colonies and it remains still to be confirmed that it takes place also in the field (see “Discussion/Open questions”).

It is known that artificial laboratory conditions can disturb normal functioning of an ant colony, including slave/slave-maker relationship. James C. Trager stressed the importance of that fact in his online statement: “In my seemingly life-long study of *Polyergu*s, I’ve observed colonies to persist for years in the field. But when brought into captive conditions, the *Formica* host workers almost always rebel, fail to raise the parasite’s brood, and begin to harass and kill off the *Polyergus* workers” (Trager, 2013a). This comment is most probably based on published cases of direct aggression between *P. lucidus* (*sensu lato*) and their *Formica* slaves (of the *F. pallidefulva* group) observed under laboratory conditions by King and Trager (2007). Mutual aggression manifested itself as biting, appendage pulling and even venom spraying. Moreover, slaves often responded aggressively to *Polyergus* queens. Slave aggressiveness escalated after several months of laboratory rearing to the point that on two occasions the queen was killed as a result of several days’ harassment. If to take into account the subsequent revision of the genus *Polyergus* Trager’s (2013b), revision of the genus *Polyergus* in one case it was a *P. longicornis* queen (killed by *F. dolosa* workers), in the second—*P. oligergus* queen (killed by *F. archboldi* workers) (King and Trager, 2007).

Devouring of enslaver brood which was observed in experimental mixed colonies of *Formica sanguinea* and wood ant workers as their untypical slaves (Czechowski, 1996) also fits well into the category ‘sabotage’, clearly leading to weakening of slave-maker colonies. For more information about this behaviour, recognised as one of the most important factors making possible self-liberation of slaves, see “Self-liberation of slaves”.

### Self-liberation of slaves

Emancipation (self-liberation) of slaves is well documented in two Palaearctic slave-makers: the blood-red ant *Formica sanguinea* (a facultative slave-maker), and the amazon ant *Polyergus rufescens* (an obligate slave-maker). Both these enslavers use species of the subgenus *Serviformica* as their slaves (e.g. Dlusskij, 1967 and Le Moli et al., 1994a, respectively). *Formica sanguinea* is additionally able, under some conditions, to enslave ant species of the subgenera *Formica* s. str. (i.e. wood ants; e.g. Marikovsky, 1963) and *Coptoformica* (Czechowski, 1977). Socially parasitic relations between *F. sanguinea* and wood ants are discussed in detail by Czechowski (1996, 2001, 2003).

#### Emancipation of slaves of *Formica sanguinea*

Even in the very early myrmecological literature (starting from the nineteenth century), there are mentions indicating that the blood-red ant *F. sanguinea* colonies containing wood ant workers as their untypical slaves may split into two homospecific parts, and that slaves may also take over the colonies of the slave-maker. Some of the *F. sanguinea* colonies experimentally mixed in the field with *F. pratensis* (by supplying them with worker pupae of the latter species) spontaneously changed with time into queenright homospecific colonies of *F. pratensis* (Forel, 1874). Forel (1928), vaguely (with no date) invoking E. Wasmann, explained these transformations by replacements of an accidentally lost *F. sanguinea* queen by a young queen of *F. pratensis* adopted by the slaves. Presently, such taking over of *F. sanguinea* nests by *F. pratensis* as an alleged slave species seems to be relatively easy to explain as this very species of wood ants has been revealed to be an occasional temporary social parasite of *F. sanguinea* colonies (at least queenless ones) (Czechowski, 2001, 2003). It is to be expected that the probability of successful takeover of a host colony by a dependent foundatrix is higher if conspecific workers and their species-specific olfactory discriminators are already present there. However, cases of taking over *F. sanguinea* colonies by enslaved wood ant workers were reported in other species of *Formica* s. str. as well (Marikovsky, 1963; Czechowski, 1996; see below). In other words, not only *F. pratensis* as a temporary social parasite of *F. sanguinea*, but also other wood ants are able to take over the colonies of that species.

Marikovsky (1963) reported that newly established colonies of *F. rufa* artificially settled in Western Siberia were often raided by *F. sanguinea* and their brood was pillaged. As a consequence, mixed colonies of *F. sanguinea* with wood ant workers came into being. However, subsequently monospecific groups of slaves escaped out of some of these mixed colonies, built their own nests in the vicinity, and recruited conspecific queens, creating thus queenright colonies of their own species. However, members of these colonies retained a friendly attitude towards workers of *F. sanguinea*, their former nestmates from still queenright *F. sanguinea* colonies. In such a way, peculiar bi-specific polydomous nest systems came into existence. It should, however, be mentioned here that the species of wood ants investigated by Marikovsky (spelled also as Marikovskij) is uncertain: it was first labelled as *F. rufa* (Marikovsky, 1963), and then as *F. polyctena* (Marikovskij, 1971). The latter identification seems to be more probable (see Czechowski, 1996).

Czechowski (1996) provided a detailed account of his observations made in the course of numerous experiments during which field colonies of *F. sanguinea* were artificially supplied with large amounts of worker pupae of several species of wood ants (*F. polyctena*, *F. rufa*, *F. aquilonia* and *F. lugubris*). These experiments were carried out in Poland and Finland in the years 1987–1996. As a result, 21 mixed colonies of *F. sanguinea* with different proportions (29–96 %) of wood ant slaves were created (Czechowski, 1996). Mass emergence of these untypical slaves in the slave-maker nests often resulted in splitting of the mixed colonies. As a rule, the majority of the slave-maker workers stayed in the old nest (supposedly the one containing the *F. sanguinea* queen), and slaves prevailed in newly established nests. Even if a colony continued to be monodomous, high differentiation of the enslaver/slave ratio in particular sectors of the extended complex nest was observed (Czechowski, 1996). Similar differentiation of the slave-maker/slave ratio is also common in mixed colonies of *F. sanguinea* and *F. cinerea* inhabiting compound nest systems typical of this host species. In such nest systems, the proportion of slaves varies from a few percent in the central part of the nest system to nearly 100 % in its peripheral parts (Dlusskij, 1967; Czechowski and Rotkiewicz, 1997; Czechowski, 1998).

Returning to the mixed colonies of *F. sanguinea* and atypical wood ant slaves created by Czechowski, during the following season, even though *F. sanguinea* workers were still present, brood of wood ants (worker larvae and pupae) appeared in some of the colonies providing an evident proof that the slaves had managed to adopt their conspecific (or related) queen(s) (Czechowski 1990, 1996). Such a situation happened in eight out of 21 mixed colonies (38 %). Only *F. polyctena* queens were adopted (in seven colonies by the conspecific slaves, and in one case by *F. rufa* slaves; Czechowski, 1996). This last case was not surprising, as in wood ants heterospecific queen adoptions in orphaned colonies happen quite frequently (e.g. Hagemann and Schmidt, 1985; Czechowski, 1996; Pisarski and Czechowski, 1994).

Takeover of an originally *F. sanguinea* colony by wood ant slaves may be temporary or definite. In four colonies in which brood of the enslaved wood ant species appeared during the next season, restitution of *F. sanguinea* (manifesting itself by the appearance of *F. sanguinea* brood) started in the following year. The remaining four colonies underwent a permanent transformation into monospecific *F. polyctena* colonies (Czechowski, 1996). Subsequent laboratory experiments revealed the existence of two mechanisms of the slave emancipation: (a) elimination of the slave-maker brood and (b) functional (not necessarily physical) queen replacement. These two processes were not independent: elimination of the slave-maker brood facilitated or even acted as a necessary condition for subsequent queen replacement (Czechowski, 1996; see below).

- *Elimination of the slave-maker brood* The striking rate at which *F. sanguinea* workers disappeared in their own field nests after the emergence of wood ant slaves, observed in a situation in which allospecific nestmate workers of these two species did not show any signs of mutual aggressiveness, strongly suggested that this phenomenon was related to the elimination of slave-maker’s brood. This hypothesis was confirmed by the results of laboratory experiments: when starvation was inflicted, mixed cultures of *F. sanguinea* and *F. polyctena* were forced to resort to brood cannibalism. In concordance with the economy of a mixed colony as the whole, younger brood was devoured first irrespectively of the species (slave vs slave-maker). As a consequence of different life histories of these two species, brood of *F. sanguinea* is usually slightly younger than brood of *F. polyctena* (Czechowski, 1996), and in the discussed laboratory experiment brood of *F. sanguinea* (larvae and relatively young pupae) was indeed generally younger than relatively aged pupae of wood ants experimentally delivered to artificial nests. During the field experiments reported above, elimination of slave-maker’s brood might also have been related to the fact that mixed colonies of *F. sanguinea* and wood ants were temporarily facing hunger as a consequence of mass emergence of callow slaves, too young to act as foragers and requiring to be fed themselves (Czechowski, 1996).
- *Queen replacement* Severe reduction of the rising generation of slave-maker workers undoubtedly made easier the next step of the slave emancipation observed in some colonies investigated in the field experiment of Czechowski (1996): the substitution of a *F. sanguinea* queen by *F. polyctena* one(s). The results of subsequent laboratory experiments (Czechowski, 1996) shed some light on other general determinants of queen replacement taking place in the mixed colonies composed of *F. sanguinea* and their atypical wood ant slaves. In particular, they revealed that splitting of the mixed colony constituted the most important factor conducive to the adoption of wood ant queens. As already told, such colony splitting into two or more parts was often observed in the field, and was experimentally simulated under laboratory conditions, too. Only queenless parts of the mixed colonies (with no *F. sanguinea* queen) were able to accept wood ant queens, both in the laboratory and in the field (Czechowski, 1996).

The experiments of Czechowski (1996) also revealed that the critical moment for the newly adopted wood ant queens comes when the colony undergoes again a fusion after a temporary splitting into a queenless and a queenright part. In two cases, single *F. polyctena* queens that had been adopted by the queenless part of the mixed laboratory colony were subsequently killed by workers from the part containing a *F. sanguinea* queen. In yet another case, 30 wood ant queens, accepted by a colony part consisting predominantly of workers of *F. polyctena* (97 %), were attacked by the queen of *F. sanguinea* which managed to kill three of them and then died, too, most probably as a result of injuries sustained in the combat with wood ant queens (Czechowski, 1996).

The above-mentioned results strongly suggest that adoption of many conspecific queens is the most important prerequisite of successful emancipation of slaves of *F. sanguinea*. Generally, in wood ants (at least in polygynous species such as *F. polyctena*), queenless groups of workers very readily adopt young conspecific queens and even related heterospecific ones (Czechowski, 1994; Pisarski and Czechowski, 1994; Yamauchi et al., 1994). During the discussed field experiments of Czechowski (1996), permanent colony takeover by *F. polyctena* most probably occurred only in those mixed colonies of *F. sanguinea* and wood ant workers which recruited numerous *F. polyctena* queens. It might also be supposed that the higher is the number of *F. sanguinea* queens present in the original colony, the more resistant is that colony against a takeover by the slave species. A single queen may not be able to survive successive, even victorious fights with several wood ant queens without serious injuries. The data on the level of gyny in *F. sanguinea* are very ambiguous, but monogyny seems to be prevalent in colonies of that ant species (see Czechowski, 1996). However, genetic data suggest that colonies of *F. sanguinea* are usually oligogynous, but functionally monogynous, i.e. they contain one egg-laying queen plus a few ‘reserve’ ones (Pamilo et al., 1978; Pamilo and Varvio-Aho, 1979). A mixed colony of *F. sanguinea* and *F. polyctena* (or similar atypical slaves) represents thus a conglomerate of ant species following different social strategies: monogyny vs high degree of polygyny. The probability of successful self-liberation of slaves from a polygynous species is particularly high, as they can recruit multiple conspecific queens, whereas *F. sanguinea* remain monogynous, and their single queen or at best few queens are much more vulnerable than multiple queens of their highly polygynous slave species.

#### Emancipation of slaves of *Polyergus rufescens*

The phenomenon of self-liberation of slaves of the obligate social parasite, the amazon ant *P. rufescens*, is the most spectacular and most advanced known form of a slave rebellion and represents the case of slave self-freeing from enslavement in the most literal sense of that word. The origin of that phenomenon may be sought in enormous numbers of enslaved workers which amazon ants accumulate in their nests, several times higher than the numbers of workers found in monospecific colonies of the host species. In *Formica fusca*, one of the most common slave species of *P. rufescens*, colony size usually does not exceed several hundred adults, rarely reaching 1,500–2,000 workers in especially large colonies (Savolainen, 1990). However, colonies of *P. rufescens* may include more than 2,000 slave-maker workers (Czechowski et al., 2012), and the proportion of slaves in colonies of various amazon ants may amount to 70–90 % of the mixed workforce (e.g. Wheeler, 1910; Talbot, 1967; Hölldobler and Wilson, 1990; Seifert, 2007; Buschinger, 2009). This means that *P. rufescens* are able to gather a dozen of thousands of slaves in their nests. Such a large number of slaves, together with the slave-maker workforce and brood and amassed slave pupae, may easily lead to overcrowding in the nest. Possible consequences of that phenomenon were revealed during the studies on a mixed colony of *P. rufescens* and *F. fusca* in the Białowieża Forest (N-E Poland) (Czechowski, 2005, 2006).

The observed colony was very large and inhabited a nest system consisting of the main nest and several small satellite nests, arranged as a semicircular at the distances of 1.6–4.4 m from the main nest entrances. At least some of the satellite nests were connected with the main nest by underground tunnels. The whole system resembled nest systems typical rather of *F. cinerea* (see e.g. Dlusskij, 1967; Czechowski and Rotkiewicz, 1997). Only the entrances of the main nest were used by *P. rufescens* (scouts and outbound and inbound raiding columns). Crews of the satellite nests consisted of small groups of *F. fusca* slaves (few dozens of workers with no brood). Sometimes, a few young sexuals and, less frequently, single workers of *P. rufescens* were seen in these satellite nests, too. During the first observation season, a dealate *F. fusca* gyne was seen twice in two different satellite nests during the period of *F. fusca* nuptial flights. In contrast to *F. sanguinea*, *P. rufescens* never bring host sexual pupae to their nest (basing on W. Czechowski’s observations of innumerable raids). Therefore, the observed gynes must have arrived from the outside and have been adopted (or at least temporarily accepted) by their enslaved conspecifics. From there, there is only one step to the emancipation of individual groups of slaves inhabiting small satellite nests. *P. rufescens* prevented such turn of events systematically raiding its own satellite nests, and carrying adult slaves present there to the main nest. No trace of mutual aggression and/or any opposition from the slaves could be observed during such raids: *F. fusca* workers were carried in a manner typical for adult nestmate transport, with the carried ant curled up beneath the carrier. These peculiar raids were starting identically as regular raids (see e.g. Dobrzańska and Dobrzański, 1960; Mori et al., 1991a, b; Le Moli et al., 1994a, b), but in the course of time they were evolving into several hours long individual activity of *P. rufescens* workers which kept running on their own in both directions between the main nest and the target satellite nest. In this way, up to 2,000–3,000 slaves were carried during a single afternoon which implies that the workers of *F. fusca* taken out of the satellite nests were systematically replaced by other (or the same) ones, coming from the main nest through underground passages. Due to their supposed function, these operations of *P. rufescens* were called the ‘integration raids’ (Czechowski, 2005).

Further observations made during the next season dotted the i’s (Czechowski, 2006; see below).The integration raids would be, most probably, fully effective to keep the integrity of the observed mixed colony, if a colony of *F. polyctena* was not present in its vicinity. A much frequented foraging route of this colony ran at a distance of 1 m from the entrances of the main *P. rufescens* nest. That route was entirely impassable for *P. rufescens* (both scouts and raiding columns). As a consequence, the area on the other side of the route was never raided during the first year of the observations. *Formica fusca* slaves were, however, able to cross freely the route of *F. polyctena*. This was not surprising, as *F. fusca* is a submissive species, well adapted to live within territories of wood ants (e.g. Savolainen and Vepsäläinen, 1989; Savolainen, 1990). Next year, the *F. polyctena* colony moved to another place, its former route disappeared, and the area behind it was now wide open for raiding by *P. rufescens*. However, still before raiding period, an increased movement of *F. fusca* slaves in this direction was observed. Not only single *F. fusca* workers, but also loose columns of several (up to 20) individuals were seen to leave the main nest of the mixed colony, to run outside the former wood ant route, and to disperse there. These expeditions resembled the so-called ‘mini raids’ of *F. fusca* slaves (see Czechowski, 2007 and also “Introduction”). However, they were undertaken only by *F. fusca*, and did not accompany the raids of the slave-maker. Only when the raids of *P. rufescens* started, they revealed a group of at least six small cryptic *F. fusca* nests scattered within an area of ca 8 m^2^, from 5.3 to 7.1 m away from the main nest of *P. rufescens* outside the former route of *F. polyctena*. Some of them contained small amounts of brood. During 11 raids observed during that season, *P. rufescens* obtained from these nests altogether about 200 worker pupae and last instar larvae. Presence of female brood implies that these *F. fusca* colonies were queenright. Prolonged stays of some raiders in the target nest were a peculiar feature of these raids. Single *P. rufescens* workers remained there even on the next day, being fully tolerated by the local *F. fusca*. Another characteristic trait of these peculiar raids consisted in gradual decline of the initial mutual aggressiveness that evolved into apparently friendly relations between the two species: allospecific workers responded to each other only by antennal palpation. At the same time, *P. rufescens* started to try to transport *F. fusca* workers to its own nest in the same way as in the course of integration raids (see above). However, as a consequence of a much less strong involvement of *P. rufescens* in this task, and a much lower readiness of *F. fusca* to accept to be transported, only about 50 adult *F. fusca* workers were successfully carried to the *P. rufescens* nest (Czechowski, 2006).

In light of these observations, it seems very probable that small cryptic colonies of *F. fusca* raided by *P. rufescens* in this peculiar way consisted of ex-slaves which had managed to break away from the enslaver colony. These ex-slaves established their own nests and adopted conspecific queens, but they apparently partly retained olfactory connection with their former mixed colony. These colonies of the newly emancipated *F. fusca* slaves would most probably gain permanent autonomy, if their *F. polyctena* neighbour had not moved away.

It may also be noted that a phenomenon belonging to the category of integration raids was signalled already by Dobrzańska (1976) in her study of a colony of *P. rufescens* with *F. cinerea* slaves. These *P. rufescens* were observed to raid (according to the author, “by mistake”) a filial nest of the mixed colony at a distance of 1.5 m from its main nest. The target nest was inhabited by *F. cinerea* slaves which were carried by *P. rufescens* to the main nest. Immediately afterwards, the majority of *F. cinerea* workers spontaneously returned to their own (filial) nest. This observation confirms the tendency of enslaved *Serviformica* workers to leave the overcrowded (?) nests of *P. rufescens*, and settle in homospecific groups in their vicinity, and the ability of *P. rufescens* to take preventive measures against that behaviour which otherwise may ultimately lead to slave emancipation.

Besides that exceptional case, J. Dobrzańska and J. Dobrzański, who studied countless raids of *P. rufescens*, never reported any further cases of capture of mature (not callow) workers from the target host species nest. However, Mori et al. (1991a, b) mentioned also the cases of capture of mature adult workers of *F. cunicularia* during three out of 38 *P. rufescens* raids observed. However, it is impossible to tell whether their observations documented capture of slaves on their way to liberation, because, unfortunately, the authors did not provide detailed information about these particular raids.

If the emancipation of enslaved workers is possible through their exodus from the nest of the slave-maker, a possible reverse mechanism may also be possible: slave emancipation may be achieved as a result of staying in the old nest when the mixed colony moves to a new place. Such periodic colony relocations are common in slave-making ants and occur, among others, to gain access to more host colonies, as recently reported for two Nearctic blood-red ant species, *F. subintegra* and *F. pergandei* (Apple et al., 2014). In the case of the Nearctic amazon ants, *P. lucidus*, such colony relocations were accompanied by the so-called emigration raids aimed at the slaves, *F. dolosa* (= *F. schaufussi*; see Trager et al., 2007) and *F. archboldi* which stayed in the old nest (Kwait and Topoff, 1983; Trager and Johnson, 1985). The biological function of these emigration raids seems to be the same as that of the integration raids observed by Czechowski (2005) in *P. rufescens*: they prevent the process of fractionation of the mixed colony. In both cases, disturbances of the process of the mixed colony unification may result in isolation of a group of the slaves, and, subsequently, in their more or less long-lasting emancipation from the social parasite.

## Discussion/Open questions

The present survey of observational and experimental data documenting various phenomena related to ‘acts of opposition’ of ant slaves against their enslavers leaves unanswered some questions which remain open for future research and reflexions.

First of all, it should be noticed that all known cases of direct hostility between allospecific individuals within mixed colonies of dulotic ant species were observed under laboratory conditions which unquestionably differ a lot from those encountered in natural nests. Therefore, a question arises whether these in vitro observations reflect faithfully the relations between slave-makers and their slaves occurring under natural conditions.

Nestmate recognition is fundamental for the integrity of social insect colonies (Crozier and Dix, 1979; Crozier and Pamilo, 1996; Sturgis and Gordon, 2012), including mixed colonies of ant slave-makers and their slaves, in which enslaved host workers do not recognise their enslavers as strangers. Only such a situation based on olfactory ‘deception’ (chemical camouflage, i.e. passive acquisition of recognition compounds, or ‘chemical mimicry’, i.e. induced biosynthesis of the proper chemical label; e.g. Lenoir et al., 2001; D’Ettorre et al., 2002; Akino, 2008; Achenbach et al., 2010; Guillem et al., 2014) ensures slave-maker’s success. For instance, D’Ettorre et al. (2002) showed that workers of *P. rufescens* reared in isolation from the mother colony developed cuticular hydrocarbon profiles closely matching those of the primary host species, which indicates the occurrence of local host adaptation in the slave-maker population.

On the other hand, the ability of slaves to recognise their homospecific nestmates allows them to act for the benefit of their maternal populations (indirectly, via weakening of the slave-maker’s colonies, and directly, via self-emancipation). Therefore, in the course of the so-called co-evolutionary arms race between social parasites and their host species (Davies et al. 1989; Lenoir et al., 2001; Tamarri et al., 2009; Delattre et al., 2012a, b), the ants from the host species may develop recognition mechanisms allowing them to identify their enslavers (for review see Delattre et al., 2012a). As a consequence, they may display agonistic behaviour more frequently in response to parasites than in response to non-parasitic local allospecifics. Such a situation was reported, among others, in *Temnothorax* species acting as potential hosts of the dulotic species *Myrmoxenus ravouxi* (Delattre et al., 2012b). Recent studies also show that host species may develop intolerance of parasite brood, which could be the first step towards slave rebellions in mixed colonies (Johnson et al., 2005, Delattre et al., 2012a).

So far, it is still not clear whether and to what degree nestmate recognition within mixed colonies of dulotic ants and their slaves depends of conditions encountered in natural and artificial nests. It is possible that distribution and blending of olfactory cues is achieved differently in Petri dishes or test tubes than in cramped and crowded natural nest spaces with organic or mineral walls saturated with established colony odour. As already told, Trager (2013a) pointed out that when colonies of *Polyergus*, which had successfully persisted for years in the field, were transferred to laboratory, the *Formica* slaves almost always failed to raise *Polyergus* brood and started to harass and kill off *Polyergus* workers (see “Sabotage”). These observations suggest some disturbances of the nestmate recognition system related to some as yet unspecified differences between physical and/or social environment experienced by ants in the field and in the laboratory. Let us remind that aggressive encounters observed within laboratory mixed colonies were not limited to skirmishes between allospecific nestmates, but they happened also between homospecific ants, both slave-makers and slaves (see “Domestic misunderstandings”). Anyway, all results concerning ant behaviour obtained under laboratory conditions, usually very simplified, ought to be approached with a certain measure of reserve. On the other hand, it is hardly imaginable to study most of the phenomena discussed here in another way than in artificial nests.

Paradoxically, all known cases of true liberation of slaves (see “Self-liberation of slaves”)—as opposed to less radical forms of the slave rebellions (“Domestic misunderstandings”, “Sabotage” and partly “Slave reproduction”)—were observed in the field. All observed cases of emancipation of slaves of the obligate slave-maker *Polyergus rufescens* belonging to a typical slave species (*Formica fusca*) were fully spontaneous from the beginning to the end. In the case of the facultative slave-maker *F. sanguinea*, slave emancipation was observed only in untypical slave species (wood ants) and was initiated artificially, either accidentally (Marikovsky, 1963) or deliberately (Czechowski, 1996). The question arises whether such latent possibility to emancipate lies dormant also in typical slaves of *F. sanguinea* (i.e. *Serviformica* species), to be expressed, too, in some specific circumstances. The first precondition for that (and, at the same time, the main difficulty) seems to be numerical predominance of slaves in the mixed colony. While in *P. rufescens* high ratio of slaves to slave-makers is a norm, the reverse is true for *F. sanguinea* (see “Self-liberation of slaves”). Although slaves of *F. sanguinea* very rarely outnumber slave-maker workers, such exceptions happen (e.g. up to 58 % of *F. fusca* and 84 % of *F. japonica*; see Czechowski, 1996), and it cannot be ruled out that typical slaves, when their proportion is high enough, might influence the *F. sanguinea* colony in some destructive way. Czechowski (1996) observed in the field a mixed colony of *F. sanguinea* in which slaves of two *Serviformica* species (*F. cunicularia* and *F. fusca*) altogether constituted 40 % of the workforce. Although the colony was queenright, there was no *F. sanguinea* female brood in the nest. In September, i.e. much later than the regular period of nuptial flights of *F. sanguinea*, only numerous males of that species (sons of slave-maker workers?) were present and their proportion reached as much as 22 % of the total number of adults of *F. sanguinea.* Two conjectures arise: either the parasite queen was infertile or something wrong was happening to her female brood. The second possibility bears much similarity to the phenomenon studied by Achenbach and Foitzik (2009) (see “Sabotage”), i.e. destroying female parasite brood by *Temnothorax* slaves enslaved by *Protomognathus americanus.* The question of existence of such phenomena in mixed colonies of *F. sanguinea* as well remains, however, to be checked by future research.

Would *Serviformica* slaves be able to adopt their conspecific queen in presence of *Formica sanguinea*? Czechowski (1996) observed that in two mixed laboratory cultures of *F. sanguinea* and their *F. fusca* slaves (a queenright one and a queenless one with 62 and 36 % share of the slaves, respectively) all experimentally introduced *F. fusca* queens were killed, and—interestingly—they were attacked mainly by *F. fusca* workers.

Another strategy which may lead to slave emancipation, i.e. selective devouring of the slave-maker brood, observed in artificially created *F. sanguinea* colonies with wood ant slaves (Czechowski, 1996), in typical (i.e. *Serviformica*) slaves seems to be even less probable than adoption of conspecific queens. As already pointed out (see “Slave emancipation”), in the conditions of starvation, younger brood is killed first. Kidnapped pupae of typical slaves are generally younger than the slave-maker’s own brood. Moreover, the ratios of natural slaves in *F. sanguinea* colonies are usually low, and during spontaneous raids the inflow of robbed pupae into the slave-maker nest is rather gradual. As a consequence, callow slaves (still inefficient and requiring care) do not represent an important burden for the mixed colony, differently than in the case of large numbers of artificially introduced wood ant slaves. However, so far nobody studied the functioning of field colonies of *F. sanguinea* artificially supplied with a surplus of typical slaves.

One may also ask if adoption of a homospecific queen represents indeed the final step (the ‘coping stone’) of a successful slave emancipation. With respect to the spectacular nature of that phenomenon, undoubtedly it does. However, if the queen is only distantly related to emancipated ex-slaves, they may gain more in terms of inclusive fitness by remaining queenless and producing their own sons than by taking care of her offspring.

In this review, we classified various phenomena documenting the resistance of slaves against their enslavers into four categories: ‘domestic misunderstandings’, slave reproduction, ‘sabotage’ and slave emancipation. These categories do not overlap, but they are not entirely discrete and at least some of them may be interrelated. For instance, it would be interesting to learn more about possible ultimate consequences of small injuries suffered by slave-makers during aggressive encounters with their slaves. As already pointed out, worker reproduction plays an essential role in the overall production of sexuals in slave-maker ants (see “Slave reproduction”). Similarly as other ants, workers of dulotic species also stay inside the nest when they are young and switch to extranidal activities as they grow older (*Formica sanguinea*: Dobrzańska, 1959; *Polyergus lucidus*: Cool-Kwait and Topoff, 1984; *Polyergus rufescens*: Le Moli et al., 1994b). However, as shown by Moroń et al. (2008, 2012), even relatively small injuries may accelerate the transition of ant workers to extranidal activities and/or induce their switching to risky tasks. Injured individuals may even abandon the nest (Hölldobler and Wilson, 1990), similarly as other moribund ant workers (Tofilski et al., 2008; Heinze and Walter, 2009). As extensively documented in many ant species, transition from intranidal to extranidal activities is as a rule accompanied by degeneration of ovaries (see “Slave reproduction”). Inverse relationship between fertility/ovarian size and engagement in extranidal activities/slave raids was also reported in the obligate slave-makers *P. americanus* (Franks and Scovell, 1983; Blatrix and Herbers, 2004; Pohl et al., 2011) and *Harpagoxenus sublaevis* (Bourke, 1988b). Therefore, the transition to extranidal and/or risky tasks induced by injuries inflicted during seemingly harmless ‘domestic misunderstandings’ with slaves may ultimately have far-reaching consequences for the slave-maker workers: it may compromise their chances to gain access to reproduction via production of male offspring. Occurrence and importance of decreased fertility of slave-makers resulting from injuries inflicted by slaves remains to be checked by future studies. However, it can be reminded that workers of *Temnothorax ambiguus* and *T. longispinosus* acting as slaves in *Protomognathus americanus* colonies were frequently observed not only to bite *P. americanus* workers, but also to drag them out of the nest (Alloway and Del Rio Pesado, 1983).

It also should be pointed out that various actions undertaken by slaves against their enslavers represent only one of the possible strategies of defence of the host species against the slave-maker. Other defence strategies involve enemy recognition, adjustment of the recognition threshold, fighting, inducible aggression, escape from the attacked nest, etc. (see Pamminger et al., 2013 and references therein). A recent experimental work (Pamminger et al., 2013) devoted to the expression of behavioural trait “slave rebellion” sensu Achenbach and Foitzik (2009) (i.e. killing of the enslaver brood), in workers of *Temnothorax longispinosus* acting as slaves of *Protomognathus americanus*, suggests that slaves employ that strategy only if other defence strategies proved to be unsuccessful. Interestingly, the lowest survival rate of slave-maker’s brood was observed in colony fragments with small numbers of slaves, and not in those with large numbers of slaves in which, theoretically, slaves could rebel most easily as a consequence of their numerical predominance. It would be interesting to check how both these matters look in other colonies of obligate social parasites.

In conclusion, we would like to express our hope that observational and experimental data discussed in this review have demonstrated convincingly that ant slaves are not as helpless as they were thought to be and that they can defend themselves against the exploitation by the slave-makers in many different ways.
